# Factors Supporting Translational, Participatory Ecology Projects—Observations from Ten Years of Collaborative Research on Invasive Fish

**DOI:** 10.1007/s00267-026-02461-w

**Published:** 2026-07-16

**Authors:** Irene Adrian-Kalchhauser, Karen Bussmann-Charran, Anouk N’Guyen, Philipp Emanuel Hirsch, Joschka Wiegleb, Lukas Bergmann, Patricia Burkhardt-Holm

**Affiliations:** 1 Institute for Fish and Wildlife Health, Department for Infectious Diseases and Pathobiology, Vetsuisse Faculty, University of Bern, Bern, Switzerland; 2 Department Aquatic Ecology, Eawag, Dübendorf, Switzerland; 3 Solothurn, Switzerland; 4 University of Applied Sciences and Arts Northwestern Switzerland, PH FHNW, Muttenz, Switzerland; 5 International Centre for Ecohydraulic Research, Department of Ecological Engineering, University of Southampton, Southampton, UK; 6 Programme Mensch-Gesellschaft-Umwelt, Department for Environmental Sciences, Science Faculty, University of Basel, Basel, Switzerland

**Keywords:** Science-policy interface, Capacity building, Aquatic invasive species, Translational participatory research, Research management, Stakeholder collaboration

## Abstract

Translational participatory research for environmental management occupies a demanding space at the interface of societal needs and academic research. Projects in this space usually face the challenge of reconciling stakeholder involvement and real-world applicability with academic career requirements such as excellence in fundamental research and the attraction of substantial research funds. Here, we share our experiences from a 12-year translational participatory research initiative on the management of invasive gobies in central Europe. We describe the project, synthesize inputs, outputs, and outcomes, and summarize conditions and practices across political, administrative, societal, institutional, team, and individual levels that we found important and beneficial in our attempts to produce both academic and applied outcomes. Our impression is that an interaction of factors across these levels created windows of opportunity, and that the team constellation and setting allowed us to seize these opportunities for the sake of both scientific progress and societal impact. By reflecting from the perspective of a multidisciplinary group of natural scientists directly engaged in such a project, we aim to contribute a personal account to social science frameworks on translational, participatory, inter- and transdisciplinary research. Our retrospective potentially can inform policy makers, funders, and institutions who seek to create enabling environments for interface research, and is helpful to fellow natural scientists who want to prepare the ground for their own translational participatory research initiatives.

## Introduction

In the broad field of natural sciences, translational participatory research activities gathering data for environmental management strategies occupy a quite challenging realm. Translational participatory research requires a careful and explicit navigation of distinct expectations of researchers, funders, and stakeholders with regards to research objectives, scientific rigor, communication, and outcomes (Lawrence et al. 2022). Society and policy are particularly interested in swift progress and decisive, locally relevant results, while the involved researchers depend on the naturally circular process of scientific investigations, and are additionally concerned with the production of deliverables supporting their scientific careers, lower funding rates of interdisciplinary grant proposals (Bromham et al. 2016), and their interest to produce generally applicable knowledge beyond local case studies. Producing knowledge that is both relevant for local environmental management and meets the needs of fundamental research is thus non-trivial, and requires targeted efforts from all parties involved (Deutsch 2025).

The field of invasion biology is a particularly rich source of translational participatory projects designed to both inform environmental management and uncover fundamental biological truths. In this research domain, the co-production of knowledge and the integration of scientific disciplines represents the state of the art (Bessert-Nettelbeck 2023). Because invasive species serve as nucleation points for novel interactions between nature and society, invasion biology is a domain in which socio-ecological knowledge generation systems are found quite frequently (Kemp et al. 2017). Projects in this realm are often translational, participatory, and may even qualify as “transdisciplinary” if they rely on the collaboration of researchers and practitioners from the private and public sectors of civil society in problem definition and knowledge production (Pohl 2021). Accounts from invasion biology projects may therefore offer valuable lessons, for example, for funding agencies with an interest to promote translational and participatory science. Such reports may also provide conservation managers with relevant insights into the temporal and group dynamics and the requirements of translational participatory research projects (Deutsch 2025). An inspection of an invasion biology project may also provide examples on how to stimulate discussions and foster mutual understanding among the environmental managers, funders, and researchers of tomorrow (Pohl 2021).

Various factors that create a favorable environment for translational and participatory projects, as well as measures of success, have been defined previously. Conducive conditions include, for example, in-person engagement and a desire to truly learn on the individual level, an awareness of “hidden” agendas at the team level, the budgeting of time for integration on the program level, the recognition of the value of applied research at the institutional level, and the willingness to navigate around technocratic hurdles on the socio-political level (Deutsch 2025). Post-hoc, the success of translational and participatory research can be estimated by examining outcomes derived from the study of real-world laboratories (Wiefek 2024), for example, scientific outputs (peer-reviewed publications or graduations), new management strategies, or policies and legal frameworks. Another post-hoc perspective on project success examines the collaborative process and the integration across disciplinary and societal boundaries (Pohl 2021), for example, by assessing the quality of stakeholder involvement, the creation of mutual trust, and the establishment of sustainable networks and new forms of cooperation. Finally, long term impacts of translational research can be estimated by examining the ability of a setting to foster learning among scientists, practitioners, and stakeholders, and thus to build capacity across sectors (Pohl 2021).

Here, we share an example of a translational and participatory research project, describe why we consider it an example of a successful project, and elaborate on conditions that we consider relevant for this success. We explicitly do not aim to engage in a formative evaluation with regard to sustainable solutions to societal problems sensu Wiefek (2024). Rather, we intend to share a peer-to-peer account and to add a perspective that might be valuable for teams engaging in translational, participatory, inter- or transdisciplinary research, for example, in ecology, conservation management, or One Health. We think that the project’s self-organized, natural-scientist-promoted nature and its sustained drive across more than a decade might add to the value of the account. We also believe such an account can be useful as a study and teaching example for students and lecturers in the field of environmental management.

The translational, participatory project under consideration unfolded in Switzerland from 2012 to 2023, and focused on invasive fish. “Non-native goby species in Switzerland—Measures to control and minimize their impact” was sparked by the detection of invasive gobies in the Rhine in Basel in 2011 during routine governmental monitoring of juvenile fish along the riverbanks. Following this information, our research group activated and invited regional stakeholders and federal administration, launched an initiative to jointly define problem areas, and then proceeded to elucidate the ecological context of the invasion and to propose and test measures to prevent further dispersal. Importantly, the specific research objectives and their relative importance were defined upfront and collaboratively with stakeholders ranging from fisheries associations to federal environmental management, and revisited during annual workshops. While we were able to *suggest* measures based on our findings, decisions on the *implementation* of measures or the implementation itself remained with the stakeholders. Our role was to *inform* relevant stakeholders about their options. Sensu Pohl (2021), this setting may even qualify as inter- and transdisciplinary, given that it was a “problem-solving setting with methodological, mostly “intermediate-scope” interdisciplinarity, with a-priori awareness for the complexity of the issue and the diversity of perceptions, aiming to link abstract with case-specific knowledge to propose solutions for a more sustainable development.”: practice and science came together from the outset for problem framing, problem analysis, and impact explorations.

**Fig. 1:**
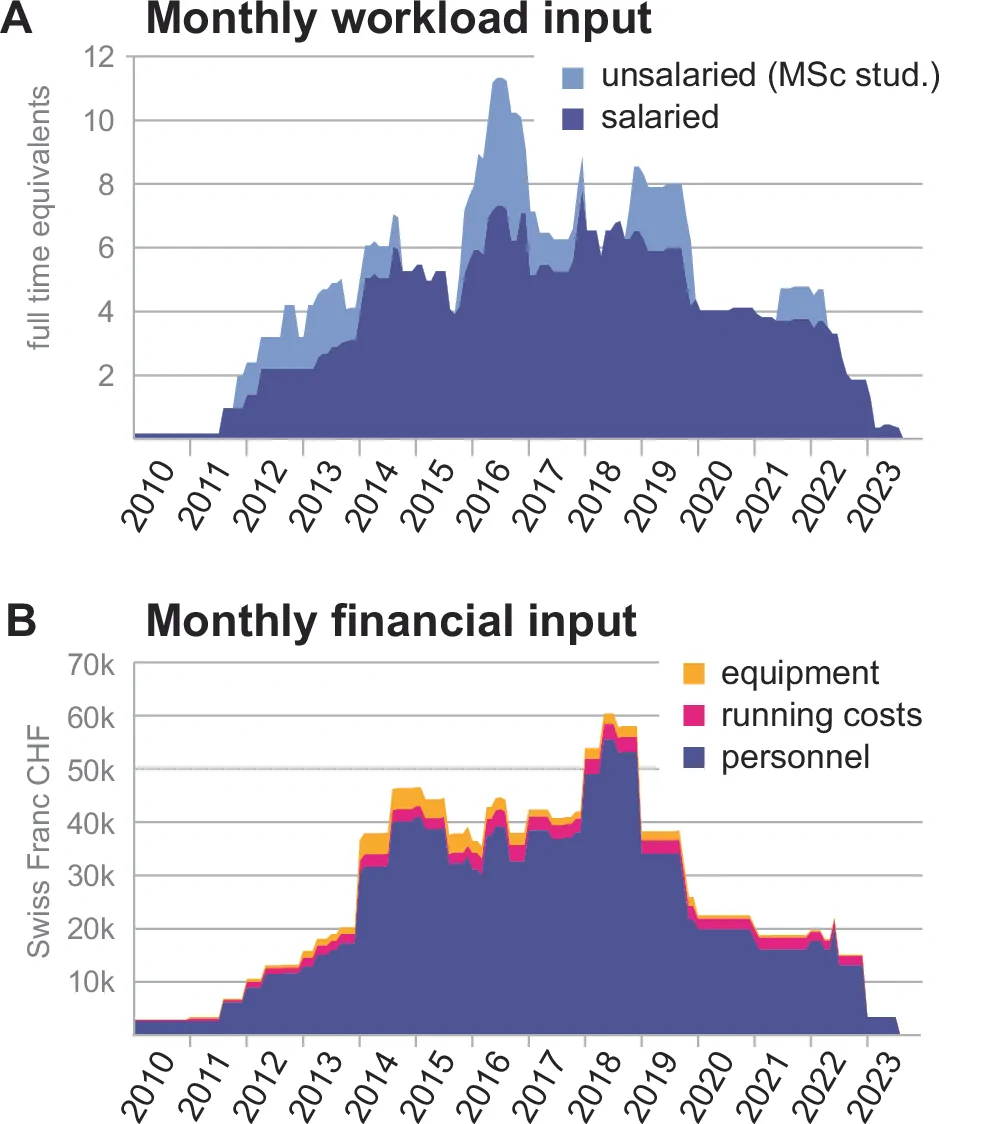
Resources consumed by the project. **A** Workload input per month follows a concave pattern over 12 years, with between 2 and 6 full time equivalents of paid personnel supported by 1–3 MSc students. **B** Financial input per month mostly follows the pattern of salaried personnel, with a non-scaling small fraction of running costs, and equipment investments peaking at 1/3rd of the project duration and trailing off in the last third of the project. During the core phase, the project required 40-50k in Swiss Francs per month, which mostly covered salaries. Non-salary components constituted 5–10% of total expenses

**Fig. 2:**
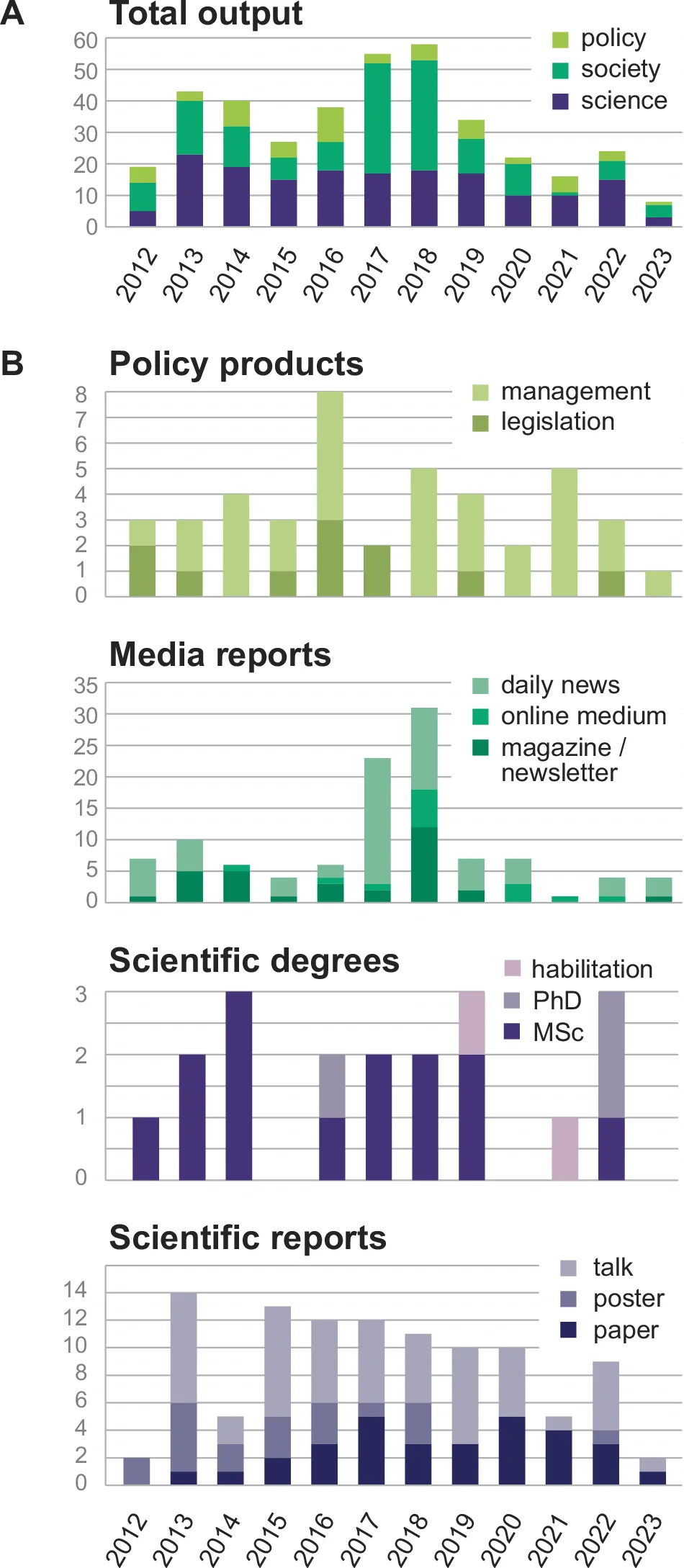
Project outputs. **A** Total output of the project across policy, society, and science (data from Supplemental Table 1). **B** Policy products, Media reports, Scientific degrees, and Scientific reports

**Fig. 3:**
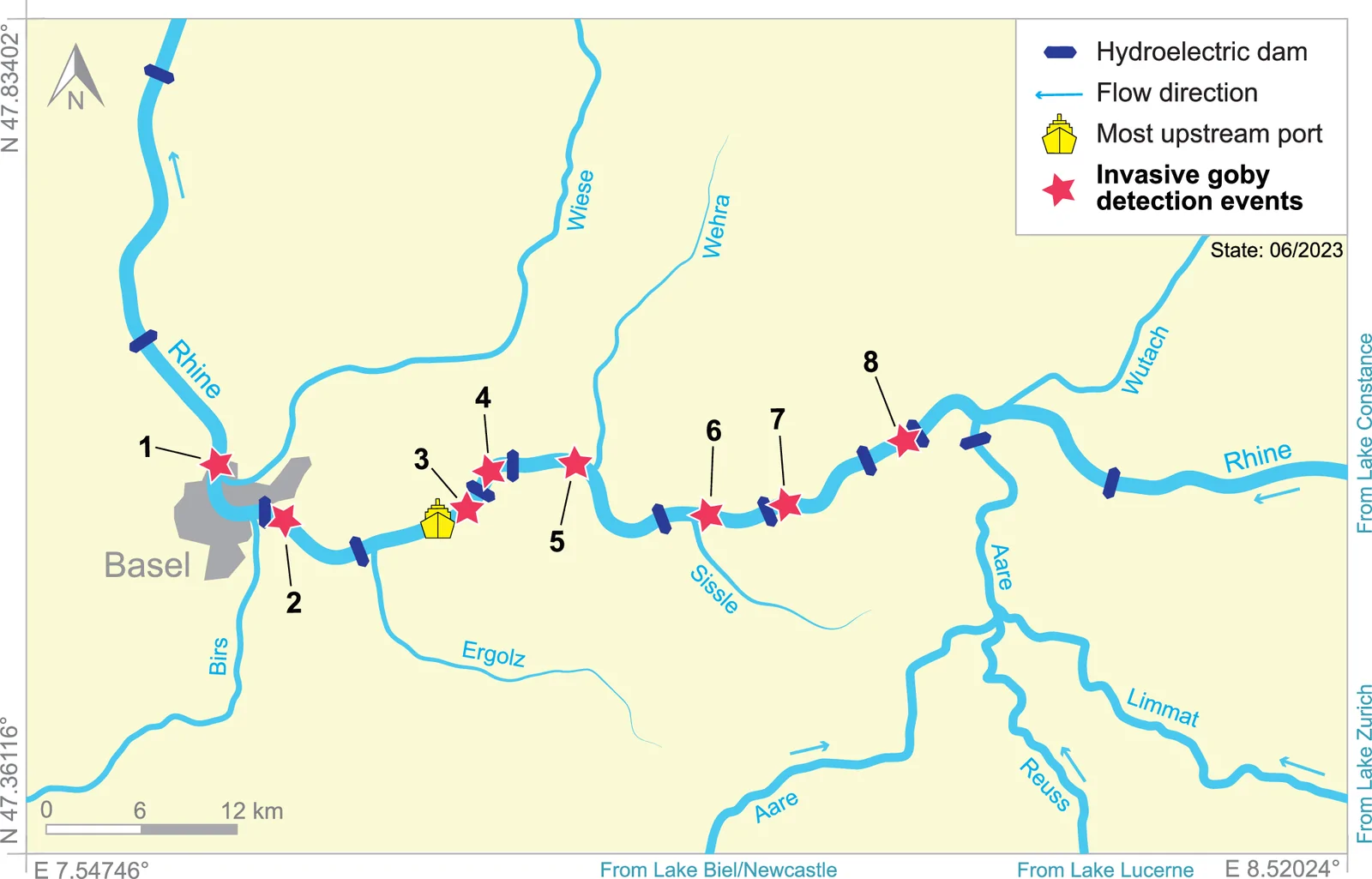
Dispersal of invasive gobies along the river Rhine. First detections upstream of the nearest hydroelectric dam (Numbered red stars) illustrate the role of the stakeholder network in monitoring the spread of invasive gobies in Switzerland. **(1) Nov 2011** governmental electrofishing campaign. **May/Jun 2012** own minnow trap fishing campaign **(2) Dec 2015** governmental monitoring campaign supported by local fishing communities. **(3) Aug 2015** governmental monitoring campaign supported by local fishing communities. **(4) Oct 2017** governmental campaign monitoring the efficiency of fish bypasses by ecobureau. (**5) Aug 2017** governmental monitoring campaign supported by local fishing communities. **(6) Sep/Nov 2018** snorkeler & local fisherman. **(7) Jun 2020** local fishermen **Jun 2022** minnow trap campaign of cantonal authorities. (**8) Jun 2023** local fishermen

Our account encompasses a description of the input in terms of human and financial resources (Fig. 1), an overview of the outcomes across three sectors policy, outreach, and research (Fig. 2), a description of the contribution of stakeholders to key results (Fig. 3) and an account of internal and external conditions - from political system to personal values of team members - that we think had a positive impact on the project (Table 1). These conditions were retrospectively and subjectively developed by all authors through repeated, open discussions and iterative revisions of a draft document across several months.

**Table 1:** Factors we consider beneficial for the described translational and participatory research project

Project-external factors (settings the project takes place in and the research team has no control over)		
Level	Factor & Description	Examples & Situation in the Goby Project
**Political level**	**Expert-friendly political culture**. Local and/or federal political environments tend to decide based on evidence and are willing to translate research results and suggestions of experts into practice.	Overall, the Swiss political culture values evidence and experts’ inputs. Local political representatives are in close touch with public opinion as well as with key stakeholders. In our case, this on occasion facilitated implementation: a prototype in-stream barrier was indeed installed. On other occasions, this prevented action: proposed measures that would impact individual freedom (e.g. boat cleaning) were considered unpopular and were thus not implemented.
	**Topicality and existing social concord**. The topic is not new; Previous or ongoing democratic processes and legislative initiatives in the subject area have already set societal goals and boundaries, clarified shared values, and highlighted knowledge gaps that guide future research needs.	Coinciding with our project, the National Biodiversity Strategy (2012) and the updated Fisheries Ordinance (2021) mandated action against invasive species, while the Federal Water Protection Law and Ordinance required the renovation of fish passes to ensure fish migration. Swiss administration therefore had prior knowledge regarding invasive species and the associated ethical and political challenges, and there was minimal dissent on the issue’s importance.
**Administrative level**	**Admin approachability**. Representatives of federal and local administrations are approachable, personally reachable, invested, and actively support the bidirectional flow of information.	Administration representatives were available for personal discussions, actively invited our input (e.g. to present at national invasive species working groups), and alerted us to new regulations or changes in processes. Research plans and proposals submitted to funding were candidly discussed prior to submission. Permits were issued fast and reliably, collaboration was efficient.
	**Decentralized decision making**. Local (approachable, see above) administrative units have substantial decision-making power regarding funding, collaboration, and project support. Time to decision is short.	In Switzerland, local authorities hold significant decision-making power as well as leeway in harm-benefit analyses and can therefore come up with workable solutions quickly. For example, we needed to sample in close vicinity to protected spawning grounds. To ensure legal compliance while enabling research, the fishery inspector personally got involved in field sampling to ensure both conservation goals and research needs were met.
	**A diverse funding landscape**. Funding opportunities across the axes of applied to basic research, of short-term activities to multi-year stipends, of private to public funders, of personal to institutional scope, and small to large sums are available.	The 10-year course of the project was supported by a colorful bouquet of grants supporting both fundamental and applied research. For instance, the Swiss National Science Foundation funded work on characterizing aspects of the round goby genome through a large personal career grant, while several smaller cantonal funds together were awarded to the host unit to study recreational boating in round goby translocation.
**Societal level**	**Consensus culture**. The societal and political culture is strongly oriented towards dialog and compromise instead of polarisation, strives to find consensus on emerging challenges, and listens to scientific evidence.	In Switzerland, a deeply rooted culture of dialog, compromise, and consensus seeking yields broadly accepted solutions, even when policies have conflicting goals. For example, the water protection law requires all waterways to be passable for all fish species by 2030, while the national strategy on invasive alien species calls for barriers to the spread of invasive species. This conflict of objectives led to solution-oriented discussions between policymakers, administrators, and scientists to strike an acceptable balance. A legal mandate to clean boats did not find support due to a high societal regard for personal autonomy, but as practical compromise, scientists, water sports clubs, and authorities collaborated to install a voluntary cleaning station.
	**High value of study object or associated resources**. The research focuses on something of high value to society (e.g. economical, recreational, symbolic), thus benefits from public attention and from a discourse for the problems and solutions at hand.	In Switzerland, the protection of water bodies is a national priority, both for their ecological importance and for their symbolic value. Significant resources are invested in aquatic conservation, such as salmon restocking programs. Public interest in water and nature-related issues is strong, which in our case lead to invited talks at water-related stakeholder organizations, media coverage, and facilitated project funding.
	**Established topical networks**. Pre-existing stakeholder networks can be accessed and offer opportunities to connect science with society. They facilitate research by providing access to locations, resources, local knowledge, and feedback on proposed measures. However, tight-knit club-like networks can also be challenging to access for outsiders.	Switzerland features a strong culture of civic participation through associations, clubs, and collaborative structures. This led to an amplification of interest in the research project and facilitated the integration of members of the public in transdisciplinary processes (N’Guyen et al., 2016, Hirsch et al., 2020). As a backdraw, individuals not organized in such associations - a phenomenon on the rise - were hard to reach. Also, dealing with networks of senior males had challenging aspects for female team members.
**Institutional level**	**Institutional commitment and supporting structures**. Translational and participatory projects benefit from systems that recognize societal impact and offer access to dedicated structures such as knowledge brokers and digital platforms. Other beneficial institutional aspects are training opportunities in communication, facilitation, and stakeholder engagement, and flexible e.g. finance frameworks that support unconventional project setups.	The unit hosting the project hosts an interfaculty master’s program (Master of Sustainable Development). The associated existing framework facilitated the contact between natural, social and economic scientists and the recruitment of students with an interest in translational and participatory projects. A stable amount of personnel funds, disposable funds, and equipment funds from the department facilitated first explorations prior to funding acquisition.
**Other**	**The Perfect Storm**. The convergence of multiple favorable conditions may create a particularly powerful momentum with a combined effect that exceeds what any single factor could achieve alone.	Three dynamically interacting factors in our project were the geographical proximity between research objects, stakeholders and researchers (essentially, an invasion happening on everyone’s doorsteps), everyone’s strong identification with the affected water bodies, and the presence of exceptionally engaged individuals in decision-making capacities on all sides.
**Political level**	**Awareness of current politics**. Knowledge of national and international strategies, policies, and regulations, an awareness of shifting priorities, and an alignment of research with both aspects increases the likelihood that research findings are of interest for policymakers and practitioners and lead to implementation.	The project was designed with careful consideration of key regulations related to invasive species and water management in Switzerland (see above). Continuous engagement of stakeholders ensured that research strategies and experimental approaches were aligned with practical policy needs. The team had a strong awareness of ongoing policy developments, and was expected to stay informed and responsive to the evolving regulatory context.
	**An existing science-policy interface**. Previous engagements at the science-policy interface and a proactive management of good connections with governmental and administrative bodies foster mutual trust and facilitate the planning and implementation of new projects.	The project built on the PI’s existing and actively maintained relationships and contacts with administration, politics and media and a high level of trust and recognition by authorities, as exemplified by multiple invitations to consult on policy matters and contributions to submission of parliamentary interventions, which in turn became drivers of the project’s visibility and influence.
**Administrative Level**	**Process awareness**. Project partners should be capable and willing to communicate explicit and implicit expectations regarding e.g. time to result, and aware of the limitations of scientific methods e.g. regarding certainty vs. probability.	A full scientific risk assessment was not possible due to lack of data, a limitation that was communicated openly. The team instead recommended a range of preventive measures and coupled them with potential consequences of non-implementation. Stakeholders then used Likert scales to estimate the probability of success of these measures, which allowed them to prioritize actions in the face of uncertainty.
**Societal level**	**Cultural awareness**. Scientists are frequently not native to the country they work in. Productive science-society exchanges therefore benefit from an awareness and discussion of cultural norms, communication styles, and institutional practices.	In the project, cultural differences and the need for approaches that would not be everyone’s primary personal choice were openly discussed and reflected. A Swiss specialty that not all team members were familiar with is the “apero” (a late afternoon get-together to network and exchange^a^) as essential tool for informal knowledge exchange and trust building.
**Institutional level**	**Institutional support**. Units doing translational and participatory research contribute to institutional reputation differently than units doing monodisciplinary science. Whether and how e.g. outreach is valued by the immediate environment, or whether DORA criteria are applied during evaluations therefore contributes to the standing and perception of units and projects.	The project team actively used available frameworks to improve the visibility and underline the relevance of their research. This included active participation in department meetings, presentation of results at colloquia, engagement as postdoc representative, or participation in lecture series and in outreach activities of the host University.
	**Teaching opportunities**. Opportunities to teach contents that are close to the research area enhances everyone’s theoretical grounding due to in-depth engagement with the literature, and also facilitates student recruitment.	The goby project was an excellent teaching example for courses about ethics, ecology, and aquatic biology in the Master of Sustainable Development considering the relevance of invasive species across social, economic, and scientific dimensions. A challenge was that teaching opportunities in other Master programs (e.g. biology) were limited.
**Project level**	**Flexible priorities and resources**. Translational and participatory projects may change course due to external inputs and events, and shifting priorities or changes in resource availability need to be expected. This requires regular strategic checkpoints and potentially adaptations of research plans.	In the project, investigations related to ballast water and boat cleaning were eventually downscaled, despite very clear relevance, partly due stakeholders’ opinions and partly due to obstacles related to data collection and accessibility. Inquiries into anti-fouling agents were eventually abandoned after preliminary planning and research due to a lack of resources.
	**Proactive stakeholder networking**. Translational and participatory projects rely heavily on networking, which thus should be acknowledged as primary task and integrated into aspects such as timelines, budgets, and expectations.	The project emphasized the importance of proactive, structured networking with stakeholders from the outset, and investments (time, space, money) were set aside to accomplish networking tasks such as writing tailored annual reports, organizing annual exchange events or participating in outreach events.
	**Community engagement beyond formal stakeholder roles**. Building genuine connections with citizen communities leads to deeper insights, unexpected collaborations, and builds trust. This requires open communication about the implications of engaging outside regular working hours (e.g., in the evenings at association meetings).	In the project, we spent time with local fishing and boating communities and sought out informal, unplanned encounters. This was not per se expected from team members, always voluntary, family commitments were explicitly and always respected, and travel expenses / time investments related to such events were reimbursed / recognized. Team members displayed an inherent interest to engage in outreach, which led to unexpected and productive collaborations on boat motor use and ballast water systems, and to sampling site access, samples, and sighting reports.
**Team level**	**Active management of team diversity**. Diversity in disciplinary backgrounds, professional expertise, and personal perspectives is a core element of translational research and, importantly, extends beyond assembling a complementary team. To harness the potential of diversity, the environment should recognize, tackle, and manage disagreements so that differences can become a source of creativity rather than of misunderstanding and frustration.	In the project, the environment was psychologically safe to voice disagreement, while team-building exercises were used to cultivate mutual appreciation for the contrasting disciplinary backgrounds. Regular opportunities for meta-level reflection on team dynamics, e.g. distinct needs in terms of support and milestones (e.g. the differing scopes and timeframes for master and PhD theses) were offered. An annual two-day team retreat dedicated time to discuss contrasting perspectives and collaboration processes.
	**Integrated career planning**. In any project, individual career development and overall project strategy should be openly discussed, and even more so in translational and participatory projects. An ongoing dialog within the team about perceived tension between career goals, publication strategies, and the demands of translational and participatory research is important.	A practical approach adopted by the project team was inclusive co-authorship. Team members invited each others’ contribution and offered opportunities for intellectual contribution and, thus, co-authorship opportunities. These invitations were tailored to personal strengths, such as preparing figures, conducting literature reviews, or drafting and integrating discussion sections.
	**Collaborative spirit**. A collaborative culture as evidenced by structured resource sharing, mutual support, the recognition of diverse perspectives, a positive attitude toward internal collaboration and joint problem-solving benefits projects in any field.	In the project, senior researchers consciously onboarded new team members e.g. through introductions to specific stakeholders. The team invested in internal communication infrastructure, such as shared contact tables, a wiki for knowledge sharing, and an annual two-day retreat. Information relevant for sub-projects of other team members, e.g. from conferences, was consciously collected and shared in labmeetings.
**Individual level**	**Curiosity, openness, and mental flexibility**. Research in any area benefits from a mindset that embraces curiosity, intellectual freedom, and adaptability. In interdisciplinary teams, however, everyone is both expert and student. Thus, a “beginner’s mind”, mental flexibility, the ability to compromise, shift perspectives, and adapt plans is particularly relevant, and a genuine interest for projects beyond one’s own is helpful.	In the project, team members were free to pursue unexpected or seemingly random questions and ideas in a playful manner. The resulting activities (e.g. field tests of DIY spawning traps) generated surprising insights or, alternatively, led to rapid changes in direction, and contributed to a culture of curiosity, experimentation, and innovation. Unfortunately, this led to staff leaving because they preferred different ways of working.
	**Transparency and awareness about career aspirations**. In line with the relevance of open discussions on career aspects, individuals need to be explicitly aware of, articulate, and on occasion defend their expectations and needs either against their own interests for yet another outreach event or the project’s requirement.	In the project, team members were encouraged to participate in discussions around career development and the challenges of publishing interdisciplinary work. This ongoing dialog created the psychological safety required to voice personal needs, and enabled the team to identify tailored solutions that balanced individual career goals with the collaborative demands of translational and participatory research.

https://togetherinswitzerland.com/swiss-apero-a-social-tradition-for-any-occasion/

## Project Characteristics and Inputs

### Team

Across 12 years, 10 natural and 2 social scientists were employed on the project, including 1 PI, 2 senior researchers, 5 postdoctoral fellows, and 4 PhD students, with an average of 3.7 full time equivalents across the entire project duration (Fig. 1). 7 of these team members were female, and 5 male. The team additionally featured, in changing composition, 24 student assistants, 2 animal care takers, 5 administrative / IT assistants, and 2 lab technicians. 31 peer-reviewed papers were first- and last-authored by researchers self-describing as aquatic ecologist, limnologist, geneticist, molecular biologist, and sustainable development experts. Co-authors often included students with an interdisciplinary master’s degree in sustainable development offered by the host institution. External co-authors had affiliations in 8 different countries (Europe and US).

### Policy and Legislation Landscape

Four notable policy works framed the project. In 1992, 20 years before the project started, Switzerland had signed the Convention on Biological Diversity (CBD) (United Nations 1992). In 2008, 4 years before the project started, the national fisheries ordinance was amended to mandate the prevention of the spread of and, if possible, eradication of alien fish and crustaceans, with federal administration in charge of coordination (SR 923.01, 2021) (Schweizerischer Bundesrat 1993). In 2012, when the project started, a national “biodiversity strategy” was adopted, stating the intention of Switzerland to conserve and promote biodiversity and ecosystem services for the economy and society (Bundesamt für Umwelt BAFU 2012). In 2016, four years into the project, a national “strategy on invasive alien species” further specified national regulations and international obligations regarding invasive alien species and identified necessary measures (Bundesamt für Umwelt BAFU 2016).

### Funding Portfolio

In total, the project resources amounted to 4.4 Mio CHF^1^, of which 3.9 Mio CHF went into salaries, 0.3 Mio CHF into consumables, and 0.2 Mio CHF into infrastructure and devices (Fig. 1B). Funds were contributed by 5 different local administrations (0.36 Mio CHF), federal administration (0.4 Mio CHF), a collaborating university unit (0.2 Mio CHF), competitive research grants (0.33 Mio CHF), funding invested from a previous foundation contribution (0.39 Mio CHF), and the unit’s own annual funds (2.74 Mio CHF). In addition, the project depended on university infrastructure (offices, laboratories, seminar rooms, fish husbandry, storage) not further monetized here.

### Stakeholders and Partners

Stakeholders that were identified and actively approached by the research team for contributions and involvement in the project included (a) practitioners such as anglers, water sports enthusiasts, boat owners, (b) industry partners such as commercial shipping firms, port authorities, and consulting firms, and (c) environmental managers such as conservation organizations and authorities responsible for management of water courses and fisheries. Stakeholder interactions relied on tools such as Futures Wheels, questionnaires, in-presence workshops, expert interviews, transdisciplinary dialogs, and scenario-driven discussions. During these interactions, stakeholders identified, prioritized, and structured research targets (for example, the initial project prioritization of negative effects on native species and population control was co-developed with fisheries association and cantonal authorities; see N’Guyen et al. 2016) and evaluated proposed measures for their perceived difficulty and effectiveness. They also contributed to data collection (for example, a network of cantonal authorities and fisher’s association was informally activated to report catch numbers of invasive gobies).

### Society & Media

Beyond the stakeholders, the research group actively communicated about project goals and outcomes through various outlets (Fig. 2C). Activities such as the annual on-site stakeholder workshops allowed for personal contact with invited media representatives, who were also proactively approached with new results and were invited to join field work. Outreach also included presentations at local fishery societies, participation in outreach for schools and the public, visits to association headquarters, contribution to a famous Swiss children’s book series (Bieri 2018) and the attendance of trade fairs in the area of fisheries and hunting.

## Project Outcomes and Outputs

The academic output of the project per March 2026 includes 32 peer-reviewed papers, 53 conference talks, 20 posters presented at conferences, 2 habilitations, 14 master theses, and 3 PhD theses (Fig. 2) along 3 major research axes described below. The project served as a stepping stone for a multitude of careers: the combination of translational, participatory project experience with sound scientific credentials allowed research team members to attain a variety of positions related to academic research and teaching, to communication positions, to applied research positions, and to positions dealing with nature-people interactions.

The knowledge gaps and research needs co-defined with various stakeholder groups shaped three axes of applied research for the project. First, the need to measure and follow the spread and the impact of the invasion led to research on novel detection methods. Detection methods evolved from standard bait-and-trap approaches (Kalchhauser et al. 2013) to the use of spawning traps (Adrian-Kalchhauser et al. 2018; N’Guyen et al. 2018), to eDNA based assays fine-tuned to the species and habitat characteristics (Adrian-Kalchhauser and Burkhardt-Holm 2016), and methods to detect predation on protected species (Lutz et al. 2020). In the end, however, anglers provided the most up-to-date reports on upstream spread (Fig. 3) - an indication for the tremendous value of their integration in the project.

Second, the need for evidence-driven decisions regarding management options while accounting for resource limitations led to impact assessments and population models. This unveiled that the impacts were difficult to predict because of their high dependence on ecosystem, time scale, and life stage (Hirsch et al. 2016a), and that resource requirements for post-introduction management were immense (N’Guyen et al. 2018). Based on these results, concepts of risk management, event probabilities, scenarios, and alternatives, and the value of precautionary management, were repeatedly discussed with stakeholders and administration.

Third, the need to design effective preventative measures led to investigations of dispersal-related traits of the invader. This revealed evidence for passive dispersal through boats, ships, and recreational activities (Bussmann et al. 2021; Adrian-Kalchhauser et al. 2016; Bussmann und Burkhardt-Holm 2020; Bussmann et al. 2022; Adrian-Kalchhauser et al. 2017), assisted dispersal by hobbyists and recreational activities (Hirsch et al. 2021), and debunked perceptions regarding the role of birds (Hirsch et al. 2018). Species traits such as egg desiccation tolerance (Hirsch et al. 2016b), personality traits (Hirsch et al. 2017), and swimming performance (Wiegleb et al. 2020; Egger et al. 2021) were also found to play a role. Results from this research axis were immediately passed on to management and administration to inform their decision making.

The three translational research axes inspired additional fundamental research projects that went beyond the stakeholder-inspired research goals, for example on goby genomics and epigenomics (Gutnik et al. 2019; Kalchhauser et al. 2013; Kalchhauser et al. 2016; Adrian-Kalchhauser und Burkhardt-Holm 2016; Adrian-Kalchhauser et al. 2017; Adrian-Kalchhauser et al. 2018; Adrian-Kalchhauser et al. 2020; Somerville et al. 2019), on the physics of swimming behaviors (Egger et al. 2021; Wiegleb et al. 2022, 2023a; Wiegleb et al. 2023b; Govindasamy et al. 2024), and on microplastic food chains (Bosshart et al. 2020).

In addition to the research publications listed above, the project produced 31 management-related products (e.g. leaflets for citizens or reports for administrative units), and 11 legislation products on the local, federal or international levels (e.g. parliamentary initiatives (Fig. 2, SI)). 6 Swiss parliamentary instruments (interpellations, postulates, motions) specifically referred to the project. The team’s activities and results led to the inclusion of five species of Black Sea gobies in the list of unwanted species in the Ordinance to the Federal Act on Fisheries (*N. melanostomus, N. kessleri, N. fluviatilis, N. gymnotrachelus, and Proterorhinus semilunaris*; (Schweizerischer Bundesrat 1993)), and contributed to the development and implementation of a national Strategy on invasive species (Bundesamt für Umwelt BAFU 2016).

Finally, the project produced numerous science communication outputs (Fig. 2) which were partly initiated by the researchers and partly by media. Since successful policy implementation relies on people’s values, which are developed early in life, we also took advantage of outreach opportunities to young citizens, for example, by contributing to one edition of a popular Swiss children’s book series on invasive species (Bieri 2018).

## Learnings and Observations

Based on its structure and its outputs, we consider our project a successful example for a translational, participatory project: the project was productive across the categories of scientific publications, career stepping stones, applied results, policy outcomes, and communication products, it featured stakeholder and administrative participation, joint reflection, and iterative adjustments, and an integration of previously unrelated elements took place (Pohl 2021). Learning and capacity building were achieved for team members and, as we believe based on their feedback and their long-term engagement, also for stakeholders, and an influence of the project on laws, regulations, and public discourse could be observed (Wiefek 2024).

We therefore asked ourselves whether we could identify conditions that potentially played a positive role during the unfolding, implementation, and completion of the project. We figured that this would improve our own understanding of the forces at work and would allow us to share our observations with other researchers in similar roles. Table 1 therefore presents 24 conditions which we believe either facilitated the project or contributed to the success we perceived. They are arranged from large-scale overarching political aspects down to the level of the individual project team member, and are combined with specific examples, similar to frameworks presented elsewhere (Deutsch 2025). Additionally, we make 8 statements of relevance to translational and participatory projects that we derived from our observations and from our experiences.

### 1. Mutual Learning Requires Time and Space

Our project went beyond including scientists from different disciplines. Sharing, aligning, and integrating field-specific skills such as molecular biology or expertise on taxonomy or behavior or on modeling, and field-specific perspectives regarding experimental design required patience and time and opportunities or spaces for knowledge transfer. This required more efforts and time resources than purely disciplinary research does, where concepts and terminology do not require upfront discussion and clarification, and also a willingness to invest into mutual understanding. An example from the project illuminating this willingness is that all junior researchers together joined a continued education on peer-to-peer coaching.

### 2. Participation in Stakeholder Engagement Activities Contributes to Individual Career Success

Building trust and establishing dialog beyond academic actors is resource-intense and emotionally taxing (N’Guyen et al. 2016; Larson and Williams 2009; Moon et al. 2015; Knapp et al. 2019). Per se, such efforts are not a requirement to achieve excellence in research or to build a career in the natural sciences. Accordingly, team members sometimes were unsure whether the resource demands of stakeholder engagement benefited or harmed their disciplinary career aspirations. From a post-hoc perspective, we are convinced that the project’s translational, participatory aspects of the project supported everyone’s subsequent career steps, since they built solid credibility regarding interface science skills and served as evidence for strong competences in managing interface research and science communication.

### 3. Diversity is a Two-edged Sword

When examining the team description above, one might consider the team constellation to some extent diverse across the dimensions of gender and discipline. We were female-led, with a male and a female postdoc as long-term team members, three female and one male PhD student, and several field- and lab assistants over time. Also, even though most researchers were natural scientists, their disciplinary backgrounds were quite distinct. This intersectionality created substantial amounts of tension and disagreement regarding e.g., experimental design, research priorities, or writing processes. Consciousness of this, and team-building excersises, thus were essential to address these topics and to promote a culture of mutual appreciation. This was for example achieved through annual research retreats with team exercises that identified everyone’s strengths, mapped complementarities, and reframed differences as valuable resources.

### 4. Open, Constructive Disagreement is a Source of Strength

As previously outlined, the project team was not always in agreement regarding research priorities, interests, or experimental design. For example, the role of aquarists in translocations of round goby was under debate for quite some time, but not everyone considered this avenue worth pursuing. However, our discussions were well-moderated and led to be productive, and team members were free to follow their ideas even without full buy-in from all group members. In the specific case of aquarists mentioned above, this led to the unexpected discovery of a substantial overlap between stakeholder groups, and thus the identification of a novel dispersal pathway (Hirsch et al. 2021).

### 5. Research Risks are Taken in an Atmosphere of Safety and Playfulness Where Failure is a Viable Option

It was easy for everyone to propose and initiate new research ideas because the decision to drop an approach or project, sometimes despite previous resource investments, was never held against individuals, and was considered a natural part of a creative process. Many initiated approaches eventually were disbanded, e.g. due to resource limitations (e.g., systematic snorkeling for the detection of goby eggs on recreational boats, boat cleaning initiative, etc.), a lack of political will (e.g., managing ballast water in inland shipping), limited consensus among relevant stakeholders (e.g. angler app for early invasion detection), changed research interests (e.g., PCR-based large-scale diet analyses), or a failure to implement reliable laboratory protocols (e.g,. round goby population genetics). The “failing is ok” mindset prevented continued investments into lost causes and helped us to focus on more promising ideas. Team members experienced an atmosphere of trust, transparency, and mutual reliance and commitment. Hiring scientifically complementary while socially fitting proactive, curious, and committed new team members was a participatory process that was taken seriously by everyone.

### 6. Institutional Design has a Major Impact

The host unit was a priori designed to produce interface content in teaching and research. Since it was administratively located in-between faculties, aspiring junior researchers had limited opportunities to teach, or supervise master students, in their respective fields. Also, the back-tracking of small-scale specialized sequencing submissions at the sequencing facility led to significant project delays. On the upside, the host unit’s interfaculty setup and focus on sustainable development was an ideal foundation to embark on a participatory and translational project.

### 7. Self-referentiality has to be Actively Navigated

Applied local research often leads to a high project-specificity of the results. Their relevance beyond the affected geographic area is often limited, there are few research groups that do comparable research, and pre-existing frameworks for (international) exchange, e.g., global organismic meetings or topical societies, are limited. We attempted to navigate this challenge by creating our own exchange opportunities (e.g,. mini-symposia and targeted meetings with specific other research groups) in addition to participating in larger overarching meetings on invasive species or gobies.

### 8. Certain Character Properties Play an Essential Role

Our team was characterized, from student assistants to PI, by an openness towards unknown and novel approaches, and a willingness to leave personal comfort zones. Another characteristic of all individuals was playfulness creativity (for example, to understand flow and drag properties of gobies, the idea to play around with 3D prints turned into a spontaneous visit of a local printing company), a trait that ensures activities are interactive, engaging and light-hearted (van Vleet und Feeney 2015). Maintaining this mindset was harder towards the end of the project (and towards the end of individual contracts), when everyone needed to focus on data analysis and publications to reach their next career goal.

## Discussion

This publication reports about a translational and participatory research project on invasion biology from the perspective of the natural scientists driving it. Our intention is to share our reflections on the conditions and practices that, in our view, facilitated the project’s productivity and impact across a number of levels. We consider these as probabilistic, non-deterministic factors: in combination, they may increase the likelihood that such a project generates both academic and societal value.

A wide array of “success factors” and “facilitating conditions” for projects in the translational, participatory, interdisciplinary, and transdisciplinary realm have been suggested previously (e.g., Pohl 2021, Wiefek 2024, Deutsch 2025). To this, we contribute the perspective of the natural scientists driving a translational project, and highlight aspects that we found particularly relevant in our constellation. For example, we acutely felt tension between career needs and project demands, in particular for early-career researchers who must balance stakeholder engagement with their output; we also very strongly perceived disciplinary disagreement as creative driver, not just as a risk to be managed; we experienced that the everyday-struggle with systemic, practical constraints (e.g. thesis supervision rights, sequencing bottlenecks) profoundly shaped the feasibility of research projects; and we surprised ourselves by gender effects when linking with male-dominated stakeholder groups or when planning field work in remote harbor areas.

The factors listed in this paper span three domains: external settings (political culture valuing evidence, approachable administrations, pre-existing societal networks, and diverse funding landscapes), internal practices (deliberate management of diversity, tolerance of failure, flexible resource allocation, proactive networking, and career-sensitive authorship strategies) and personal dispositions (curiosity, openness, and the willingness to step outside disciplinary and personal comfort zones). Our impression is that interactions across all these domains created “perfect storms of opportunity,” where momentum emerged not from a single factor but from their convergence. This echoes frameworks in the social sciences (Deutsch 2025) and grounds them in lived experience.

We would appreciate an extension of our observations into linked but distinct fields that may have different drivers or perspectives. For example, the terrestrial stakeholder-, management- and policy field focuses less on habitats and more on species (groups), which leads to a fragmentation of knowledge (Heiderich et al. 2024), which in turn may potentially impact integration. Also, our exchanges with neighboring countries suggest that the centralization of decision structures results in fundamentally different challenges and opportunities. We’d like to encourage research teams in these areas to share their observations in a similar manner.

Some of the listed factors are out of the range of influence for a research team, either completely, or within the timeframe of a research project, such as societal culture or political environments. Nevertheless, being explicit about such conditions can help teams anticipate opportunities, mitigate constraints, make intentional strategic choices and capitalize on strongholds. Informal feedback from participants in a continued education event on One Health (CAS One Health, University of Bern, November 2025, Basic Module 2) supports this idea: They found the idea of awareness in the absence of an ability to influence highly useful. We therefore would like to end with the recommendation to fellow researchers in translational and participatory research to discuss our and other’s suggestions for facilitating conditions at strategic meetings throughout a project.

## Supplementary information


supplementalfile1-20250505


## Data Availability

No datasets were generated or analysed during the current study.

## Footnotes

1 applicable exchange rate: 1 CHF ~ 1 EUR ~ 1.2 USD
